# Quality and outcomes framework achievement and unplanned admissions for cardiovascular disease

**DOI:** 10.1186/s12913-025-13227-1

**Published:** 2025-10-03

**Authors:** Bo Hou, Sian Reece, Rachael H. Moss, Jamilla Hussain, Tom Lawton, Michael McCooe, Kuldeep Sohal, Sohail Abbas, Tim Doran, Trevor Sheldon, Josie Dickerson

**Affiliations:** 1 Bradford Institute for Health Research, Temple Bank House, Bradford Royal Infirmary, Duckworth Lane, Bradford, BD9 6RJ UK; 2 Division of Surgery and Interventional Science, University College London, Charles Bell House, 43-45 Foley Street, London, W1W 7JN UK; 3 Leicester Medical School, University of Leicester, George Davies Centre, Lancaster Rd, Leicester, LE1 7HA UK; 4 Department of Health Sciences, University of York, Seebohm Rowntree Building, Heslington, York YO10 5DD UK; 5 NHS West Yorkshire Integrated Care Board, White Rose House, West Parade, Wakefield, WF1 1LT UK; 6 Wolfson Institute of Population Health, Queen Mary University of London, Charterhouse Square, London, EC1M 6BQ UK

**Keywords:** Unplanned hospital admissions, Quality and outcome framework, Health inequalities, Cardiovascular disease, Primary care

## Abstract

**Background:**

Unplanned hospital admissions are costly and disproportionately affect people who are socioeconomically disadvantaged and from an ethnic minority group. A national primary care pay-for-performance scheme, the Quality and Outcomes Framework (QOF), was introduced in England in 2004 to financially incentivise general practices to meet a range of performance indicators, but the QOF’s impact on unplanned hospital admissions remains unclear. We examined the association between unplanned hospital admissions for cardiovascular disease (CVD), individual-level characteristics and achievement of key QOF indicators for CVD at the patients’ registered general practice.

**Methods:**

This study used the Connected Bradford dataset, which links individual-level primary and secondary care data. Our analytical sample included 508,977 patients registered with a Bradford District general practice from 2017 to 2019.

Logistic regression was used to estimate associations between achievement of relevant QOF indicators and unplanned admissions for cardiovascular diseases, adjusting for individual-level differences in age, sex, ethnicity, socioeconomic status and pre-existing health conditions.

**Results:**

Significantly reduced odds of unplanned CVD hospital admissions were associated with attending a practice with higher achievement rates for QOF indicators relating to atrial fibrillation management (OR 0.97, *p* < 0.001), diabetes management (OR 0.98, *p* = 0.002), and smoking cessation (OR 0.98, *p* = 0.038). Conversely, increased odds of unplanned admission were associated with higher achievement for QOF indicators relating to antiplatelet or anticoagulation medication (OR 1.06, *p* < 0.001) and blood pressure control for diabetic patients (OR 1.02, *p* = 0.03). Individual-level characteristics significantly associated with increased risk of unplanned admission included living in the most deprived fifth of neighbourhoods (OR 2.00, *p* < 0.001) and having Pakistani ethnicity (OR 1.65, *p* < 0.001). Primary care diagnoses of hypertension (OR 1.79, *p* < 0.001), diabetes (OR 1.56, *p* < 0.001), chronic cardiac disease (OR 2.79, *p* < 0.001), and stroke (OR 1.6, *p* < 0.001) were all statistically significant and associated with higher odds of unplanned admissions for CVD.

**Conclusions:**

We found mixed evidence for an association between practice-level QOF achievement and unplanned hospital admissions for CVD. There were large ethnic and socioeconomic inequalities in unplanned admissions for cardiovascular disease. Supporting general practices to appropriately improve their achievement of key cardiovascular disease related QOF indicators and reducing socioeconomic inequalities might likely reduce the number of unplanned hospital admissions.

**Supplementary Information:**

The online version contains supplementary material available at 10.1186/s12913-025-13227-1.

## Introduction

Unplanned hospital admissions are unexpected and urgent admissions to hospital and represent a growing burden for health settings across the world [1]. They are costly, create uncertainty for managers of health systems and can be unpleasant for patients and their families [2]. Unplanned hospital admissions are a key proxy indicator of health inequalities in healthcare in numerous countries [3, 4].

Cardiovascular disease (CVD), such as ischaemic heart disease, stroke and peripheral vascular disease, are major causes of burden on individuals and healthcare services across the world [5]. Ischaemic heart disease is the leading cause of years of life lost and has remained one of the main causes of mortality over the past several decades in England [6]. Many of these conditions are considered ambulatory care sensitive conditions because hospital admissions for these conditions can be prevented through interventions in primary care [7].

High quality, evidence-based clinical care, alongside timely treatment of acutely ill patients in primary care can reduce hospital admissions [8]. Furthermore, there is evidence that effective primary and secondary prevention in primary care is associated with reduced hospital admission rates, such as for stroke [9]. However, measuring quality of primary care is challenging, as quality of care incorporates many different and complex facets including: being safe, effective, person-centred, timely, efficient and equitable [10].

The Quality and Outcomes Framework (QOF) was introduced in England in 2004 as a national primary care pay-for-performance scheme to financially incentivise general practices for meeting a range of performance indicators that are primarily related to the management of chronic conditions [11].

Early studies at a national level have found that the use of QOF indicators reduced disparities between general practices in the delivery of incentivised processes of care [12], and was associated with reduced hospital admissions for ambulatory care sensitive conditions, such as chronic obstructive pulmonary disease [13]. There are however, mixed results for some chronic conditions such as coronary heart disease and diabetes [14–17] and no overall effect on mortality was found [18].

Most existing studies evaluating QOF outcome data are ecological studies that use aggregated data at general practice level rather than individual-level data. One of the key issues for ecological studies is ecological fallacy, precluding robust patient- level inferences [19]. This issue is exacerbated where there are large variations in the distributions of the outcome, such as variations in hospital admissions between different populations of general practices in England. A recent study showed that factors related to local area socioeconomic characteristics accounted for more variation in admissions for incentivised conditions than achievement of QOF indicators [20]. Most studies are national studies that used Hospital Episode Statistics [9, 21]. It is not clear to what extent QOF relates to unplanned hospital admissions for CVD in an ethnically diverse and socioeconomically deprived population. Additionally, most empirical studies evaluating unplanned admissions using QOF data were conducted in the early stages of the programme [16], which has since undergone significant changes.

To address these gaps, we used a large individual-level dataset with linked routine primary and secondary data from an ethnically diverse city with high levels of deprivation to examine the association between unplanned hospital admissions for CVD, individual-level characteristics and achievement of key QOF indicators for CVD at the patients’ registered general practice.

## Methods

### Study setting and design

Bradford is a large city in the North of England with over half a million people in its population. It has high levels of socioeconomic deprivation and an ethnically diverse population, and its inner-city areas experience some of the worst health inequalities in England [22].

This study used a bespoke dataset that contained linked primary and secondary care data at individual patient level from the Connected Bradford programme. Connected Bradford is a data linkage programme that provides information on health and different aspects of patient level data [23]. Data linkage between primary and secondary care data was conducted using pseudonymised NHS numbers. The dataset for this analysis was extracted and built in July 2023.

A cohort study design was used. Our analytical sample included patients who registered with a Bradford general practice before 1 st January 2017 and remained an active patient as of 31 st December 2019. This excluded people who left, joined or were otherwise removed from the practice lists during this period.

### Outcomes

Unplanned hospital admissions data were obtained from Secondary Uses Service data from two main acute trusts in Bradford District for the time period of 2017–2019. We focused on the ambulatory care sensitive conditions that were the most common causes of unplanned hospital admissions in Bradford [7], linked by common pathophysiological mechanisms and clinical risk factors, including congestive heart failure, hypertension, angina, stroke, transient ischaemic attack and peripheral vascular disease. Relevant ICD-10 codes used for coding these conditions are listed in Appendix S1. The outcome of this analysis is whether the patient had an unplanned hospital admission for any of these conditions during this period.

### Exposure

We identified the QOF indicators most likely to affect the risk of unplanned admissions for CVD. We included clinical QOF indicators related to atrial fibrillation (AF), coronary heart disease (CHD), heart failure (HF), hypertension (HYP), chronic kidney disease (CKD), diabetes (DM), stroke (STIA) and smoking (SMOK). We selected indicators that more closely related to the active clinical management of conditions or the risk factors for such conditions.

As the incentive scheme operates at the practice level, we used QOF indicators measured at general practice level. The QOF data at general practice level was accessed through the NHS digital website [24]. In our analysis, for the achievement of each QOF indicator, the proportion of the eligible general practice population that achieved the indicator was used. The list of QOF indicators selected is summarised in the main results table and described in full in Table 1. An additional table containing information on the variation in the achievement of key QOF indicators across general practices is disclosed in Appendix S2.

**Table 1:** QOF indicators included in this analysis

Indicator label	QOF indicator	Indicator description
AF Register	AF006	The percentage of patients with atrial fibrillation in whom stroke risk has been assessed using the CHA2DS2-VASc score risk stratification scoring system in the preceding 12 months (excluding those patients with a previous CHADS2 or CHA2DS2-VASc score of 2 or more)
AF + CHADS Vasc > = 2 + Anticoagulated	AF007	In those patients with atrial fibrillation with a record of a CHA2DS2-VASc score of 2 or more, the percentage of patients who are currently treated with anticoagulation drug therapy
BP Measure	BP002	The percentage of patients aged 45 or over who have a record of blood pressure in the preceding 5 years
CVD Statin	CVD-PP001	In those patients with a new diagnosis of hypertension aged 30 or over and who have not attained the age of 75, recorded between the preceding 1 April to 31 March (excluding those with pre-existing CHD, diabetes, stroke and/or TIA), who have a recorded CVD risk assessment score (using an assessment tool agreed with the NHS CB) of ≥ 20% in the preceding 12 months: the percentage who are currently treated with statins
CHD Anticoag/platelet	CHD005	The percentage of patients with coronary heart disease with a record in the preceding 12 months that aspirin, an alternative anti-platelet therapy, or an anti-coagulant is being taken
CHD BP ≤ 140	CHD008	The percentage of patients aged 79 years or under, with coronary heart disease, in whom the last blood pressure reading (measured in the preceding 12 months) is 140/90 mmHg or less.
CHD BP ≤ 150	CHD009	The percentage of patients aged 80 years or over, with coronary heart disease, in whom the last blood pressure reading (measured in the preceding 12 months) is 150/90 mmHg or less.
HF DiagConfirm	HF002	The percentage of patients with a diagnosis of heart failure (diagnosed on or after 1 April 2006) which has been confirmed by an echocardiogram or by specialist assessment 3 months before or 12 months after entering on to the register
HF ACEi/ARB	HF003	In those patients with a current diagnosis of heart failure due to left ventricular systolic dysfunction, the percentage of patients who are currently treated with an ACE-I or ARB
HF Betablocker	HF004	In those patients with a current diagnosis of heart failure due to left ventricular systolic dysfunction who are currently treated with an ACE-I or ARB, the percentage of patients who are additionally currently treated with a beta-blocker licensed for heart failure
HYP BP ≤ 140	HYP003	The percentage of patients aged 79 years or under, with hypertension, in whom the last blood pressure reading (measured in the preceding 12 months) is 140/90 mmHg or less.
HYP BP ≤ 150	HYP007	The percentage of patients aged 80 years or over, with hypertension, in whom the last blood pressure reading (measured in the preceding 12 months) is 150/90 mmHg or less.
DM ACEi	DM006	The percentage of patients with diabetes, on the register, with a diagnosis of nephropathy (clinical proteinuria) or micro-albuminuria who are currently treated with an ACE-I (or ARBs)
DM EduProg	DM014	The percentage of patients newly diagnosed with diabetes, on the register, in the preceding 1 April to 31 March who have a record of being referred to a structured education programme within 9 months after entry on to the diabetes register
DM BP ≤ 140	DM019	The percentage of patients with diabetes, on the register, without moderate or severe frailty in whom the last blood pressure reading (measured in the preceding 12 months) is 140/80 mmHg or less.
DM HbA1c ≤ 58	DM020	The percentage of patients with diabetes, on the register, without moderate or severe frailty in whom the last IFCC-HbA1c is 58 mmol/mol or less in the preceding 12 months.
DM HbA1c with frailty + ≤ 75	DM021	The percentage of patients with diabetes, on the register, with moderate or severe frailty in whom the last IFCC-HbA1c is 75 mmol/mol or less in the preceding 12 months.
DM Statin	DM023	The percentage of patients with diabetes, on the register, and a history of CVD (excluding haemorrhagic stroke) who are currently treated with a statin.
STIA Anticoag/Platelet	STIA007	The percentage of patients with a stroke shown to be non-haemorrhagic, or a history of TIA, who have a record in the preceding 12 months that an anti-platelet agent, or an anti-coagulant is being taken
STIA BP ≤ 140	STIA010	The percentage of patients aged 79 years or under, with a history of stroke or TIA, in whom the last blood pressure reading (measured in the preceding 12 months) is 140/90 mmHg or less.
STIA BP ≤ 150	STIA011	The percentage of patients aged 80 years or over, with a history of stroke or TIA, in whom the last blood pressure reading (measured in the preceding 12 months) is 150/90 mmHg or less.
Smoke Status	SMOK002	The percentage of patients with any or any combination of the following conditions: CHD, PAD, stroke or TIA, hypertension, diabetes, COPD, CKD, asthma, schizophrenia, bipolar affective disorder or other psychoses whose notes record smoking status in the preceding 12 months
Smoke OfferTx (SMOK004)	SMOK004	The percentage of patients aged 15 or over who are recorded as current smokers who have a record of an offer of support and treatment within the preceding 24 months

Where necessary, the QOF indicator used was an average of the three years from 2017 to 2019 at general practice level. Several QOF indicators (AF001, CHD001, HF001, HYP001, DM017, CKD005, STIA001, OB002) related to whether general practices established and maintained registers of patients with related health conditions were not included in the analysis because all practices included had achieved these indicators and so there was no variation between practices. Smoking related indicators were related to whether smoking status had been ascertained not whether the patient was a current smoker. Also, only active general practices throughout this period were considered.

### Covariates

Patient level demographic information was determined from primary care data. We harmonised and mapped ethnicity codes in primary and secondary care to 2011 census categories [25]. The quintiles for the Index of Multiple Deprivations (IMD) 2019 at Lower Layer Super Output Areas were used to proxy for socioeconomic status [26]. Patient level CVD related pre-existing health conditions (hypertension, diabetes, chronic cardiac disease and stroke) before 2017, were included from patient primary care records using OpenSafely code lists [27].

### Statistical analysis

Logistic regressions were used to estimate associations between achievement of QOF indicators and unplanned admissions for CVD. One fully adjusted model was run. This model included all relevant QOF indicators measured at general practice level and individual risk factors including age, sex, ethnicity, quintiles for the IMD, and individual health conditions. Odds ratios (OR) are reported with 95% confidence intervals and *p*-values.

Clustered standard errors at GP practice level were used to address the clustering nature of the data [28]. Complete case analyses were conducted. Stata (17, StataCorp LLC, College Station, TX) and R studio (R version 4.1.2) were used to extract and analyse the data.

### Sensitivity analysis

Two level random intercept models were also used to account for the clustered nature of the population by general practices [29], see Appendix S3. To address potential bias of excluding patients who may have died during the study period, the same analysis was repeated on a younger sample of patients < = 80 years old who have a lower risk of mortality, See section S4.

## Results

### Descriptive statistics on cohort characteristics

The analytical sample included 508,997 patients registered at a Bradford general practice between 1st January 2017 and 31st December 2019, and a total of 70 general practices. There were 3,436 spells of unplanned admissions for CVD, which represents 0.7% of the analytical sample. Table 2 describes the cohort characteristics.

**Table 2:** Descriptive statistics of cohort characteristics (*n* = 508,997)

Variable list	*N*	Mean	SD
Age	508,997	40.6	22.6
	***N***	**Percentages (%)**	
Gender			
Male	255,942	50.0	
Female	253,055	50.0	
Ethnicity			
White British	230,209	45.2	
Other White	21,313	4.2	
Pakistani	96,872	19.0	
Other Asian groups (non-Pakistani)	26,220	5.2	
Black, African, Caribbean or Black British	5,988	1.2	
Mixed or multiple ethnic groups	6,499	1.3	
Other ethnic groups	7,367	1.5	
Unknown/Refused	114,529	22.5	
Index of Multiple Deprivations 2019 in quintiles			
Most deprived	210,923	48.3	
Second most deprived	88,410	20.2	
Third most deprived	52,605	12.0	
Fourth most deprived	47,644	10.9	
Least deprived	37,533	8.6	
Pre-existing conditions			
Hypertension	70,280	13.8	
Diabetes	38,147	7.5	
Chronic cardiac disease	21,116	4.2	
Stroke	5,944	1.2	
Unplanned admissions for CVD between 2017–2019			
No	505,561	99.3	
Yes	3,436	0.7	

45.2% of the total study population were White British. The Pakistani group was the largest ethnic minority group at 19.0% of the population. 22.5% of patients refused to state their ethnicity or the ethnicity information was missing. Almost half (48.3%) of the study population lived in the most deprived quintile of IMD and 8.6% in the least deprived IMD quintile. The prevalence of pre-existing hypertension, diabetes, chronic cardiac disease and stroke were 13.8%, 7.5%, 4.2% and 1.2% respectively. The demographics of the study population are broadly representative of the Bradford population for the same time period, see Appendix S5.

### Associations between achievement of key QOF indicators and unplanned admissions for cardiovascular diseases

**Fig. 1:**
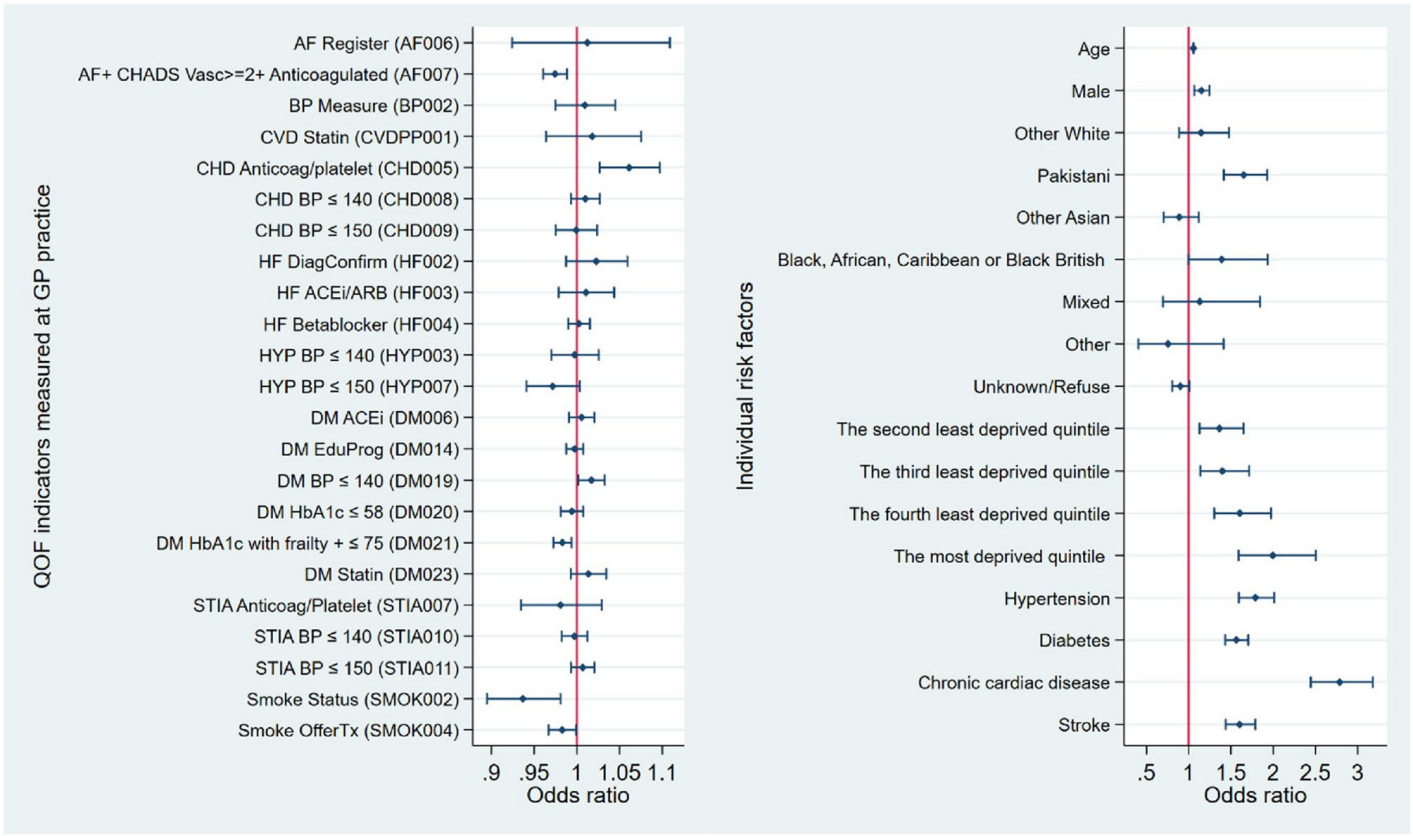
Associations between achievement of key QOF indicators and unplanned admissions for cardiovascular diseases - plotted from the fully adjusted model and presented in two panels for clarity

The results of associations between achievement of key QOF indicators and unplanned admissions for CVD are presented in Table 3. Figure 1 shows the estimated odds ratios from the fully adjusted model. A reduced odds ratio for achievement of QOF indicators was expected in models, as this indicated the lower unplanned admissions for CVD associated with a higher percentage of patients in a practice achieved relevant QOF indicators.

**Table 3:** Associations between achievement of key QOF indicators and unplanned admissions for cardiovascular diseases, logistic regressions

Having an unplanned admission for CVD	Fully adjusted model, odds ratios	95% CI	*P* value
QOF Indicators measured at GP practice			
AF Register (AF006)	1.01	[0.92,1.11]	0.793
AF + CHADS Vasc > = 2 + Anticoagulated (AF007)	0.97	[0.96,0.99]	<0.001
BP Measure (BP002)	1.01	[0.97,1.05]	0.604
CVD Statin (CVDPP001)	1.02	[0.96,1.08]	0.524
CHD Anticoag/platelet (CHD005)	1.06	[1.03,1.10]	<0.001
CHD BP ≤ 140 (CHD008)	1.01	[0.99,1.03]	0.245
CHD BP ≤ 150 (CHD009)	1.00	[0.98,1.02]	0.947
HF DiagConfirm (HF002)	1.02	[0.99,1.06]	0.212
HF ACEi/ARB (HF003)	1.01	[0.98,1.04]	0.520
HF Betablocker (HF004)	1.00	[0.99,1.02]	0.706
HYP BP ≤ 140 (HYP003)	1.00	[0.97,1.03]	0.856
HYP BP ≤ 150 (HYP007)	0.97	[0.94,1.00]	0.078
DM ACEi (DM006)	1.01	[0.99,1.02]	0.467
DM EduProg (DM014)	1.00	[0.99,1.01]	0.617
DM BP ≤ 140 (DM019)	1.02	[1.00,1.03]	0.030
DM HbA1c ≤ 58 (DM020)	0.99	[0.98,1.01]	0.387
DM HbA1c with frailty + ≤ 75 (DM021)	0.98	[0.97,0.99]	0.002
DM Statin (DM023)	1.01	[0.99,1.03]	0.200
STIA Anticoag/Platelet (STIA007)	0.98	[0.93,1.03]	0.430
STIA BP ≤ 140 (STIA010)	1.00	[0.98,1.01]	0.714
STIA BP ≤ 150 (STIA011)	1.01	[0.99,1.02]	0.319
Smoke Status (SMOK002)	0.94	[0.89,0.98]	0.005
Smoke OfferTx (SMOK004)	0.98	[0.97,1.00]	0.038
Individual risk factors			
Age	1.06	[1.05,1.06]	< 0.001
Male	1.15	[1.06,1.24]	<0.001
White British	1.00	-	-
Other White	1.15	[0.89,1.48]	0.294
Pakistani	1.65	[1.41,1.93]	<0.001
Other Asian	0.89	[0.70,1.12]	0.314
Black, African, Caribbean or Black British	1.39	[1.00,1.94]	0.050
Mixed	1.13	[0.69,1.84]	0.622
Other	0.76	[0.40,1.41]	0.381
Unknown/Refuse	0.90	[0.80,1.01]	0.076
The least deprived quintile	1.00	-	-
The second least deprived quintile	1.36	[1.13,1.65]	0.001
The third least deprived quintile	1.40	[1.14,1.72]	0.001
The fourth least deprived quintile	1.60	[1.30,1.97]	< 0.001
The most deprived quintile	2.00	[1.59,2.50]	< 0.001
Hypertension	1.79	[1.59,2.01]	<0.001
Diabetes	1.56	[1.43,1.71]	< 0.001
Chronic cardiac disease	2.79	[2.44,3.18]	< 0.001
Stroke	1.60	[1.44,1.79]	< 0.001
Observations	430,368		
Pseudo *R*^2^	0.22

In the fully adjusted model, achievement of appropriate anticoagulation management for high risk patients with atrial fibrillation (AF Anticoag (AF007)) (OR 0.97, *p* < 0.001), diabetic control (DM HbA1c with frailty + ≤ 75 (DM021)) (OR 0.98, *p* = 0.002), record of smoking status (Smoke Status (SMOK002)) (OR 0.94, *p* = 0.005) and offer of smoking cessation therapy for patients who smoke (Smoke OfferTx (SMOK004)) (OR 0.98, *p* = 0.038) were statistically significant and showed reduced odds. A 1% increase in underlying achievement rate of the practice population for AF Anticoag (AF007) was associated with a 3% reduction in the odds of an unplanned admission for CVD, holding other variables constant.

However, two QOF indicators were statistically significant and were associated with increased odds. They were indicators related to the management of patients with high risk cardiovascular heart disease with anticoagulation or antiplatelet therapy (CHD Anticoag/platelet (CHD005)) (OR 1.06, *p* < 0.001) and adequate blood pressure control for diabetic patients (DM BP ≤ 140 (DM019)) (OR 1.02, *p* = 0.03).

For individual risk factors, age increased odds of unplanned admissions for CVD (OR 1.06, *p* < 0.001). Compared to females, males had higher odds of having CVD unplanned admissions (OR 1.15, *p* < 0.001). Compared to the White British, those documented as being of Pakistani ethnicity (OR 1.65, *p* < 0.001) showed highest odds of CVD related unplanned admissions followed by those documented as being of Black, African, Caribbean or Black British ethnicity (OR 1.39, *p* = 0.05). Compared to the least deprived quintile of deprivation, there was a statistically significant and positive gradient shown, with each higher quintile of IMD showing increased odds of having unplanned admissions for CVD– the second least deprived quintile (OR 1.36, *p* = 0.001), the third least deprived quintile (OR 1.40, *p* = 0.001), the fourth least deprived quintile (OR 1.60, *p* < 0.001) and the most deprived quintile (OR 2.00, *p* < 0.001). Primary care diagnoses of hypertension (OR 1.79, *p* < 0.001), diabetes (OR 1.56, *p* < 0.001), chronic cardiac disease (OR 2.79, *p* < 0.001), and stroke (OR 1.6, *p* < 0.001) were all statistically significant and associated with higher odds of unplanned admissions for CVD.

In the fully adjusted model, except for primary care diagnoses of health conditions, the variables that had the largest size of odds ratios were living in the most deprived quintile (OR 2.00, *p* < 0.001) and Pakistani ethnicity (OR 1.65, *p* < 0.001). Our main findings remained robust in all sensitivity analyses.

## Discussion

### Summary

We found mixed evidence on associations between QOF achievement at general practice level and unplanned hospital admissions for CVD in Bradford population. Patients who attended a practice with a greater achievement of key QOF indicators related to management of high-risk patients with atrial fibrillation (AF Anticoag), diabetes management (DM HbA1c ≤ 75), smoking status (Smoking Status) and being offered smoking cessation therapy (Smoke OfferTx) had reduced odds of unplanned hospital admissions. However, we also found that attending a practice with higher rates of achievement for indicators relating to management of patients with high-risk cardiovascular disease (CHD Anticoag) and blood pressure control for patients with diabetes (DM BP ≤ 140) was associated with increased odds of unplanned hospital admission. This may be due to underlying complex interactions between risk factors included in this analysis and partly reflect multifaceted risk factors related to unplanned hospital admissions for CVD. These associations remained significant after controlling for individual-level differences in age, sex, ethnicity, socioeconomic status and pre-existing health conditions.

Importantly this analysis also highlighted large health inequalities in the occurrences of unplanned admissions for CVD. Ethnicity and deprivation were independently and significantly associated with unplanned hospital admissions for CVD in our analysis. Compared to the White British group, even after accounting for individual socioeconomic status and pre-existing health conditions, individuals from Pakistani ethnicity still had much higher odds of unplanned hospital admissions for CVD. There was also an increased odds of unplanned admissions for CVD with each decrease in IMD quintiles compared to the most deprived quintile after adjusting for risk factors in our model.

### Strengths and limitations

We were able to use a large individual-level dataset with linked primary and secondary care data that covers the Bradford population. Our results found mixed evidence to support the achievement of QOF indicators in reducing unplanned admissions for CVD in a large, deprived and ethnically diverse city, and lastly, our exploration examined more recent data.

The quality of routine data for research purposes is often limited [7, 30]. We had access to data from two main hospitals, however patients from the general practice lists included in this study may have attended different hospitals for unplanned care treatment, which subsequently we would not have identified in this dataset. We were also only able to capture risk factors available in the routine data. In addition, we only considered a sample of stable patients registered with a Bradford general practice in the whole study period, partly due to the lack of access to the national mortality dataset. If the probability of death differs according to exposure, our results may be affected. Although our results remained consistent and robust using a younger sample who are at a lower risk of mortality.

There are also caveats to acknowledge with the use of QOF indicators as a reflection of clinical care received by patients. The QOF is not a mandatory framework and does not reflect the full extent of clinical practice guidelines for the management of such clinical conditions and as such may not reflect the care received by patients. Due to data limitation, we used practice level QOF indicators instead of patient specific information on conformance to QOF indicators in our analyses, this may mask the true relationship between the effect of the degree of conformance in the care of individual patients on the probability that a patient would experience an unplanned hospital admission for cardiovascular disease. There are also complexities on reported QOF statistics, such as exceptions related to each indicator. Exception reporting is a mechanism that allows GP practices to exclude eligible patients from an indicator’s denominator for various reasons including informed dissent, recent registration with the GP or contradiction for a specified intervention [31]. Exception reporting may be used to artificially increase GP practices reported achievement [32]. The level of exception reporting might be higher GP practices in more deprived areas [33]. Therefore it adds an extra layer of complexity to QOF.

Furthermore, the variations in QOF indicators were generally small between the practices included in the analysis. During this period, some QOF indicators were discontinued, and a few practices were closed.

Some QOF indicators relate to similar activities in distinct patient groups, for example CHD008 (percentage of patients aged 79 years or under with coronary heart disease with blood pressure ≤ 140/90 mmHg) and CHD009 (percentage of patients aged 80 years or over with coronary heart disease with blood pressure ≤ 150/90 mmHg). In these cases, we included both indicators in the analysis because they are likely to capture two different population groups, one of which is more likely to have complex comorbidities [34]. Other QOF indicators may also correlate with each other and have complex interactions. The selection of QOF indicators in this analysis was decided a priori based on clinical theory and practice. The aim of our analysis is to explore associations rather than causal.

### Comparisons with existing literature

Previous ecological studies on hospital admissions for CVD and achievement of QOF indicators found mixed results [14–16]. Our results supported previous ecological studies that showed mixed results on QOF indicators achievement and reductions in unplanned hospital admissions for CVD [35].

A systematic review that described primary care factors that influenced unplanned secondary care use in Organisation for Economic Cooperation and Development (OECD) countries found that age, socioeconomic status, chronic disease and multi-morbidity were important individual-level factors [36]. Ethnicity, distance to hospital, rurality, lifestyle and access to primary care have also been identified as important risk factors for avoidable hospital admissions [37–39]. Particularly, level of deprivation showed strong positive associations with unplanned hospital admissions at a population level [9, 16, 40]. Our results also confirmed strong social gradients in unplanned hospital admissions for CVD. Patients from the largest ethnic minority group in this study, those of Pakistani ethnicity, had higher odds of unplanned hospital admissions compared to White British patients, and this remained when accounting for socioeconomic deprivation and comorbidities. This highlights a need for further research to understand the underlying causes of this ethnic disparity in health.

### Implications for research and or practice

Using real-world evidence, our study demonstrated the powerful and independent association of deprivation and ethnicity with unplanned CVD admissions. The findings are particularly timely, aligning with the current direction of the NHS in England, where newly established Integrated Care Systems (ICSs) have a statutory duty to reduce health inequalities. The mixed results for QOF achievement suggest that to make a meaningful impact on unplanned admissions and reduce inequalities, ICSs cannot simply rely on traditional performance metrics such as the QOF.

 Instead, our work underscores the need for system-wide strategies that address the wider social determinants of health, in alignment with national initiatives that aim to address these inequalities. For example, Core20PLUS5 is a national NHS England approach to inform action to reduce healthcare inequalities at both national and system level. The people with highest CVD risk are the same people who also suffer from wider health and social care inequalities. The link between Government’s major conditions strategy and Core20PLUS5 framework would be vital. It is important to not underestimate the targeted resources this work requires based on equity principles. Action at a population level is needed as well as a focus on individual behaviours and primary care.

Supporting general practices to improve their achievement against key CVD related QOF indicators, such as active management of atrial fibrillation and diabetes mellitus and increasing provision for smoking cessation services may likely reduce the number of unplanned hospital admissions. Further research should look at other cohorts and see whether there is a theoretical reason to make these indicators special or if it is due to the specifics of our cohort. One advantage of the Connected Bradford data platform is that it can track the performance of GP practices over time. This would allow for future time-series and longitudinal study analyses that may help us better understand the complex association between QOF and health outcomes.

## Supplementary Information

Supplementary Material 1.

## Data Availability

The data that support the findings of this study are available through the Connected Bradford data platform. But restrictions apply to the availability of these data to the public. Application of access to the data platform can be made through an Expression of Interest form to the Connected Bradford project team at Bradford Institute for Health Research.

## Abbreviations

QOF: Quality and Outcome Framework
CVD: Cardiovascular disease
CI: Confidence Interval
IMD: Index of Multiple Deprivation
OECD: Organisation for Economic Cooperation and Development
OR: Odds Ratio
