# Supplementary material for: Quality and outcomes framework achievement and unplanned admissions for cardiovascular disease
Source: BMC Health Serv Res. 2025 Oct 3;25:1297. doi: 10.1186/s12913-025-13227-1 (PMC12495635; doi:10.1186/s12913-025-13227-1)
Supplement: Supplementary file 1 — Supplementary Material 1. [file 12913_2025_13227_MOESM1_ESM.docx]

Supplementary Information

Table S1 ICD-10 codes used for defining unplanned hospital admissions for cardiovascular diseases

| ACSC name | ICD10 code | Comments |
| --- | --- | --- |
| Congestive heart failure ^[[1]](#footnote-1)^ | I110 Hypertensive heart disease with (congestive) heart failure  I50 Heart failure J81 Pulmonary oedema | Principal diagnosis only; Exclude main operative procedures with OPCS4 codes of K0,K1,K2,K3,K4,K50,K52,K55,K56,K57, K60,K61,K66,K67,K68,K69,K71 |
| Angina | I20 Angina pectoris  I240 Coronary thrombosis not resulting in myocardial infarction  I248 Other forms of acute ischaemic heart disease  I249 Acute ischaemic heart disease, unspecified | Principal diagnosis only; Exclude cases with main operative procedure OPCS4 codes of A, B,C, D, E, F, G, H, I, J, K, L, M, N, O, P, Q, R, S, T, V, W, X0, X1, X2, X4, X5 |
| Hypertension | I10 Essential (primary) hypertension  I119 Hypertensive heart disease without (congestive) heart failure | Principal diagnosis only; Exclude cases with main operative procedure OPCS4 code of K0, K1, K2, K3, K4, K50, K52, K55, K56, K57, K60, K61, K66, K67, K68, K69, K71 |
| Stroke and TIA^[[2]](#footnote-2)^^[[3]](#footnote-3)^ | 'I63', 'I636', 'I634', 'I631','I633', 'I630','I635','I632','I639','I61','I613','I614','I611', 'I610', 'I612', 'I615', 'I616','I619','I638','I618','I64','I64X', 'I62', 'I621', 'I629', 'G453', 'G451' ,'G452', 'G458', 'G459', 'G45', 'G454' ,'G450' |  |
| Peripheral vascular  disease^[[4]](#footnote-4)^ | 'I73', 'I738', 'I739', 'I731' |  |

S2: Variations in achievements of key QOF indicators across general practices (N=70) in this analysis

| QOF indicators | Mean (%) | Std. dev. | Min (%) | Max (%) |
| --- | --- | --- | --- | --- |
| af006 | 98.7 | 1.3 | 94.0 | 100.0 |
| af007 | 90.8 | 5.0 | 74.4 | 100.0 |
| bp002 | 90.7 | 3.1 | 85.3 | 98.3 |
| cvdpp001 | 99.6 | 1.2 | 91.7 | 100.0 |
| chd005 | 95.8 | 2.9 | 87.9 | 100.0 |
| chd008 | 83.3 | 6.4 | 62.3 | 100.0 |
| chd009 | 88.9 | 3.5 | 76.9 | 100.0 |
| hf002 | 95.3 | 2.3 | 89.7 | 100.0 |
| hf003 | 99.0 | 2.4 | 79.2 | 100.0 |
| hf004 | 92.3 | 5.1 | 76.9 | 100.0 |
| hyp003 | 75.3 | 6.5 | 56.9 | 89.9 |
| hyp007 | 86.2 | 3.8 | 78.8 | 100.0 |
| dm006 | 93.8 | 7.2 | 69.2 | 100.0 |
| dm014 | 93.9 | 7.4 | 59.1 | 100.0 |
| dm019 | 74.9 | 9.8 | 51.6 | 92.5 |
| dm020 | 66.4 | 8.5 | 47.2 | 80.6 |
| dm021 | 91.1 | 6.6 | 60.0 | 100.0 |
| dm023 | 93.8 | 4.8 | 73.9 | 100.0 |
| stia007 | 97.3 | 2.3 | 90.0 | 100.0 |
| stia010 | 79.8 | 8.1 | 60.1 | 100.0 |
| stia011 | 89.8 | 6.3 | 54.5 | 100.0 |
| smok002 | 95.7 | 1.8 | 91.9 | 99.7 |
| smok004 | 93.6 | 4.6 | 79.9 | 100.0 |

S3: The multilevel approach

If there is sufficient clustering between general practices, the multilevel model can allow us to explore the variance at practice (level 2) and patient level (level 1). To assess extent of clustering in our data structure, the intra-class coefficient (ICC) was used(37). The ICC coefficient after each model was listed in the table S2 below. In the null model, intra-class correlation was 3.5%, which was suggesting that 3.5% of variation in unplanned admissions for CVD was between GP practices. The rest of variations in the outcome were due to patient level differences within general practices i.e. clustering of individuals in practices.

The ICC stayed close to zero in the full model. In other words, this suggests that there was little variation in unplanned admissions for CVD in our data structure that was due to differences between practices. Thus, the benefits of a multilevel approach compared to a one level model are limited in this case, as there was little variation at the general practice level. This may be partly due to QOF indicators being collected at practice level as opposed to individual-level.

Nevertheless, two-level random intercept multilevel models were also run. No appreciable differences were found. This is available upon request.

Table S3 Intra-class correlation coefficients for each multi-level model

|  | Intra-class correlation coefficient |
| --- | --- |
| Null model, outcome only | 0.035 |
| Fully adjusted model | 0.014 |

S4. Study Population and Cohort Selection Details

The Connected Bradford dataset is a comprehensive database containing linked primary care, secondary care, and other data for the entire population of the Bradford district. This is possible as all general practices in Bradford use the same clinical systems provider (TPP). For this study, data from each practice was updated monthly.

Our analysis required a stable patient cohort to assess the association between practice-level QOF achievement for the study period and unplanned hospital admissions over a fixed period. Therefore, our inclusion criteria, applied via a SQL query, selected all patients who were continuously registered with a Bradford GP practice throughout the entire study period (2017–2019). This approach necessarily excluded patients who deregistered from a Bradford practice during this period.

We acknowledge the important limitation that this exclusion could introduce selection bias. Due to the dynamic nature of large-scale electronic health records, although we have our data extract, the original data snapshot from 2017 is no longer accessible, which prevents a direct baseline comparison between our final cohort and all patients who were excluded. Furthermore, we did not have access to national ONS mortality data, making it impossible to definitively distinguish between patients who moved away and those who died. While our linked secondary care data identified approximately 200 deaths during this period, this is likely an underestimation. We have detailed these data constraints as a limitation in the main manuscript's Discussion section.

To directly address the potential for bias relative to mortality – excluding patients who may have died and thus de-registered from the GP system, we conducted a robust sensitivity analysis on a subgroup where the primary source of the bias—death—is much less frequent. The primary driver for this bias is mortality, which is strongly age-dependent. We therefore repeated our fully adjusted model on a younger cohort of patients (aged <80 years), where the risk of mortality during the study period would be substantially lower. The results of this sensitivity analysis confirmed that our main findings remained consistent and robust, providing strong evidence that our conclusions are not primarily driven by survivorship bias.

Table S4

| Having an unplanned admission for CVD | Fully adjusted model, odds ratios | 95% CI | Fully adjusted model  age<=80 | 95% CI |
| --- | --- | --- | --- | --- |
| **QOF Indicators measured at GP practice** | | | |  |
| AF Register (AF006) | 1.01 | [0.92,1.11] | 1.02 | [0.92,1.14] |
| AF+ CHADS Vasc>=2+ Anticoagulated (AF007) | 0.97 | [0.96,0.99] | 0.98 | [0.96,0.99] |
| BP Measure (BP002) | 1.01 | [0.97,1.05] | 1.02 | [0.98,1.06] |
| CVD Statin (CVDPP001) | 1.02 | [0.96,1.08] | 1.04 | [0.98,1.11] |
| CHD Anticoag/platelet (CHD005) | 1.06 | [1.03,1.10] | 1.06** | [1.02,1.10] |
| CHD BP ≤ 140 (CHD008) | 1.01 | [0.99,1.03] | 1.01 | [0.99,1.02] |
| CHD BP ≤ 150 (CHD009) | 1.00 | [0.98,1.02] | 0.99 | [0.97,1.02] |
| HF DiagConfirm (HF002) | 1.02 | [0.99,1.06] | 1.01 | [0.98,1.05] |
| HF ACEi/ARB (HF003) | 1.01 | [0.98,1.04] | 1.01 | [0.98,1.04] |
| HF Betablocker (HF004) | 1.00 | [0.99,1.02] | 1.00 | [0.99,1.02] |
| HYP BP ≤ 140 (HYP003) | 1.00 | [0.97,1.03] | 1.00 | [0.97,1.03] |
| HYP BP ≤ 150 (HYP007) | 0.97 | [0.94,1.00] | 0.97 | [0.94,1.00] |
| DM ACEi (DM006) | 1.01 | [0.99,1.02] | 1.01 | [0.99,1.02] |
| DM EduProg (DM014) | 1.00 | [0.99,1.01] | 1.00 | [0.99,1.01] |
| DM BP ≤ 140 (DM019) | 1.02 | [1.00,1.03] | 1.02 | [1.00,1.03] |
| DM HbA1c ≤ 58 (DM020) | 0.99 | [0.98,1.01] | 0.99 | [0.98,1.01] |
| DM HbA1c with frailty + ≤ 75 (DM021) | 0.98 | [0.97,0.99] | 0.98 | [0.97,0.99] |
| DM Statin (DM023) | 1.01 | [0.99,1.03] | 1.02 | [0.99,1.04] |
| STIA Anticoag/Platelet (STIA007) | 0.98 | [0.93,1.03] | 0.98 | [0.93,1.03] |
| STIA BP ≤ 140 (STIA010) | 1.00 | [0.98,1.01] | 1.00 | [0.98,1.01] |
| STIA BP ≤ 150 (STIA011) | 1.01 | [0.99,1.02] | 1.01 | [0.99,1.02] |
| Smoke Status (SMOK002) | 0.94 | [0.89,0.98] | 0.93 | [0.89,0.98] |
| Smoke OfferTx (SMOK004) | 0.98 | [0.97,1.00] | 0.98 | [0.97,1.00] |
| **Individual risk factors** | | | |  |
| Age | 1.06 | [1.05,1.06] | 1.06 | [1.06,1.07] |
| Male | 1.15 | [1.06,1.24] | 1.18 | [1.07,1.29] |
| White British | 1.00 | - | 1.00 | - |
| Other White | 1.15 | [0.89,1.48] | 1.00 | [0.70,1.44] |
| Pakistani | 1.65 | [1.41,1.93] | 1.49 | [1.21,1.83] |
| Other Asian | 0.89 | [0.70,1.12] | 0.89 | [0.69,1.14] |
| Black, African, Caribbean or Black British | 1.39 | [1.00,1.94] | 1.44 | [0.92,2.24] |
| Mixed | 1.13 | [0.69,1.84] | 1.30 | [0.74,2.30] |
| Other | 0.76 | [0.40,1.41] | 0.92 | [0.50,1.70] |
| Unknown/Refuse | 0.90 | [0.80,1.01] | 0.91 | [0.79,1.04] |
| The least deprived quintile | 1.00 | - | - |  |
| The second least deprived quintile | 1.36 | [1.13,1.65] | 1.47 | [1.25,1.73] |
| The third least deprived quintile | 1.40 | [1.14,1.72] | 1.46 | [1.22,1.76] |
| The fourth least deprived quintile | 1.60 | [1.30,1.97] | 1.82 | [1.48,2.25] |
| The most deprived quintile | 2.00 | [1.59,2.50] | 2.31 | [1.86,2.88] |
| Hypertension | 1.79 | [1.59,2.01] | 1.85 | [1.61,2.11] |
| Diabetes | 1.56 | [1.43,1.71] | 1.57 | [1.41,1.76] |
| Chronic cardiac disease | 2.79 | [2.44,3.18] | 3.12 | [2.73,3.56] |
| Stroke | 1.60 | [1.44,1.79] | 2.07 | [1.80,2.39] |
| Observations | 430,368 |  | 410374 |  |
| Pseudo *R*^2^ | 0.22 |  | 0.199 |  |

S5: Bradford population statistics

QOF 2019-2020 prevalence [data](https://app.powerbi.com/view?r=eyJrIjoiMDZiMmI2MzEtMWVjZC00YTVlLWI5NjEtMTNkODM3M2M0NDk3IiwidCI6IjUwZjYwNzFmLWJiZmUtNDAxYS04ODAzLTY3Mzc0OGU2MjllMiIsImMiOjh9) from NHS Digital indicated that the prevalence rates of hypertension, diabetes, atrial fibrillation, and stroke and transient ischaemic attack were 13.4%, 8.9%, 1.6%, 1.7%, respectively. In addition, the latest 2021 census ethnicity [data](https://www.ons.gov.uk/peoplepopulationandcommunity/culturalidentity/ethnicity/bulletins/ethnicgroupenglandandwales/census2021) showed the White British and the Pakistani ethnic group accounted for 56.7% and 25.5% respectively of the population living in the Bradford Local Authority.

1. [2.3.i Unplanned hospitalisation for chronic ambulatory care sensitive conditions - NHS Digital](https://digital.nhs.uk/data-and-information/publications/statistical/nhs-outcomes-framework/august-2021/domain-2---enhancing-quality-of-life-for-people-with-long-term-conditions-nof/2.3.i-unplanned-hospitalisation-for-chronic-ambulatory-care-sensitive-conditions) [↑](#footnote-ref-1)
2. [Emergency readmissions to hospital within 30 days of discharge by diagnosis : indirectly standardised percent trends broken down by sex (I02041) - NHS Digital](https://digital.nhs.uk/data-and-information/publications/statistical/compendium-emergency-readmissions/current/emergency-readmissions-to-hospital-within-30-days-of-discharge-by-diagnosis) [↑](#footnote-ref-2)
3. [Meeting the ambition of measuring the quality of hospitals' stroke care using routinely collected administrative data: a feasibility study - PMC (nih.gov)](https://www.ncbi.nlm.nih.gov/pmc/articles/PMC3723302/) [↑](#footnote-ref-3)
4. <https://www.datadictionary.nhs.uk/Covid19PRA/Peripheral_Vascular.html> [↑](#footnote-ref-4)
